# Supplementary material for: Multi-Omics Analyses Reveal Mitochondrial Dysfunction Contributing to Temozolomide Resistance in Glioblastoma Cells
Source: Biomolecules. 2023 Sep 19;13(9):1408. doi: 10.3390/biom13091408 (PMC10526285; doi:10.3390/biom13091408)
Supplement: Supplementary file 1 [file biomolecules-13-01408-s001.zip › Supplementary figures S1-S9.pdf]

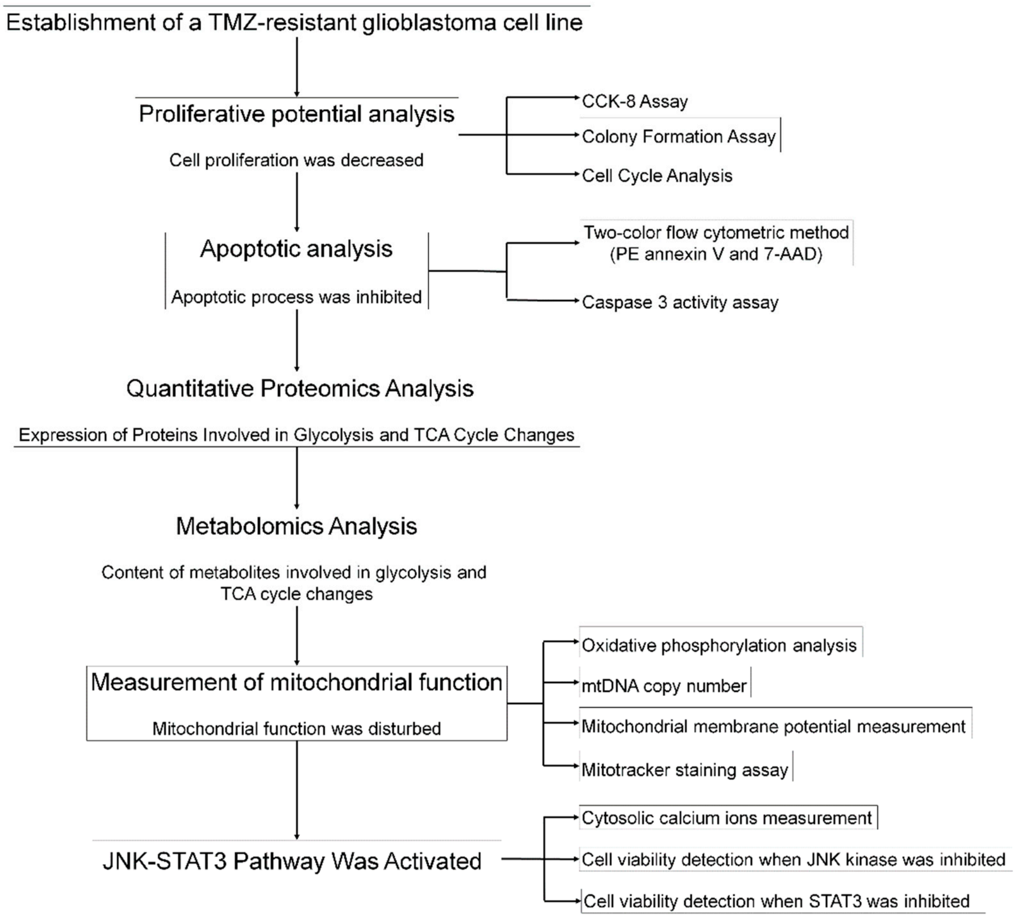

Figure S1. Demonstration of the workflow for the investigation of TMZ-resistance mechanism.

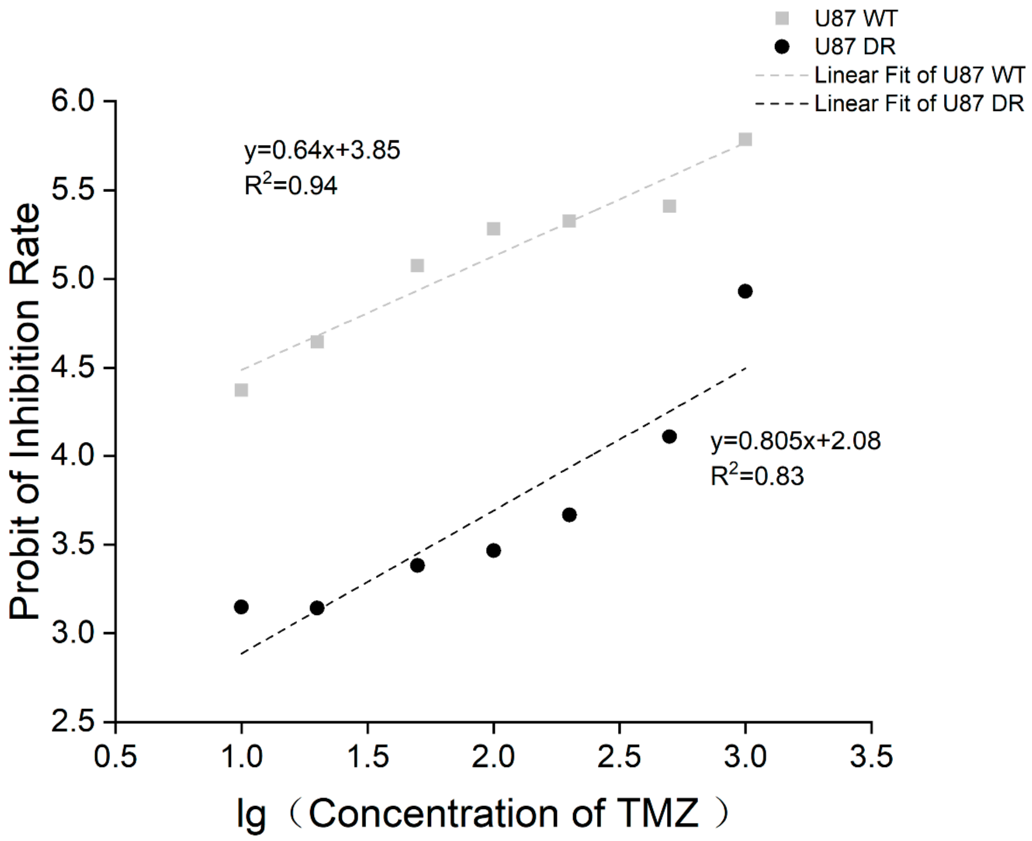

Figure S2. Calculation of IC 50 (the half maximal inhibitory concentration) of TMZ. The logarithm of the compound concentration is linearly relative to the probit converted from the inhibition rate. When the inhibition rate was 50%, probit was 5, IC50 was calculated according to the linear equations of U87 WT cells and U87 DR cells, respectively.

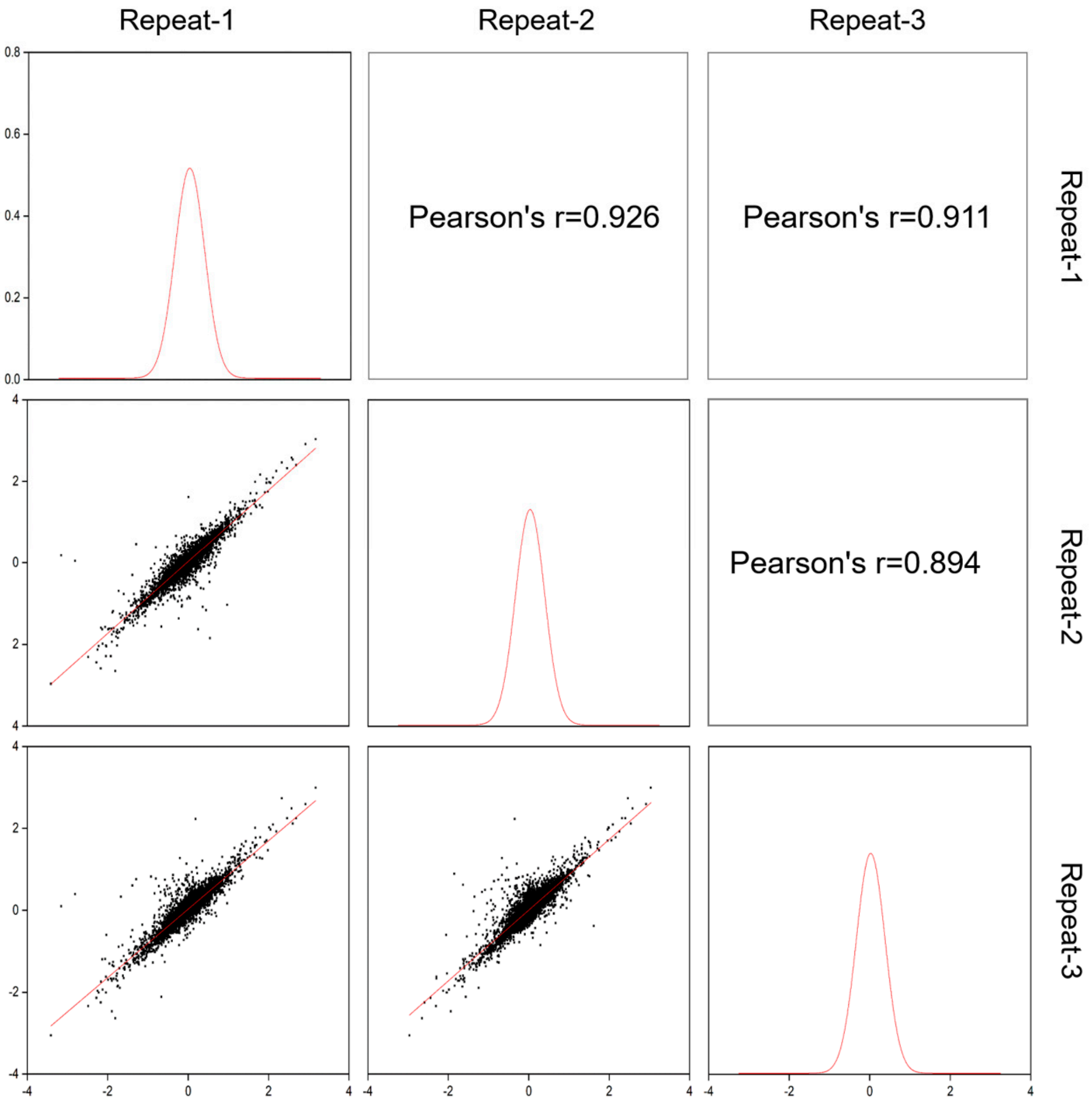

Figure S3. Quality control of proteomics data. A normal distribution of proteomics data was shown using the normality test and distribution curve. Correlation analysis of each group was shown using Pearson's correlation coefficient.

a

b

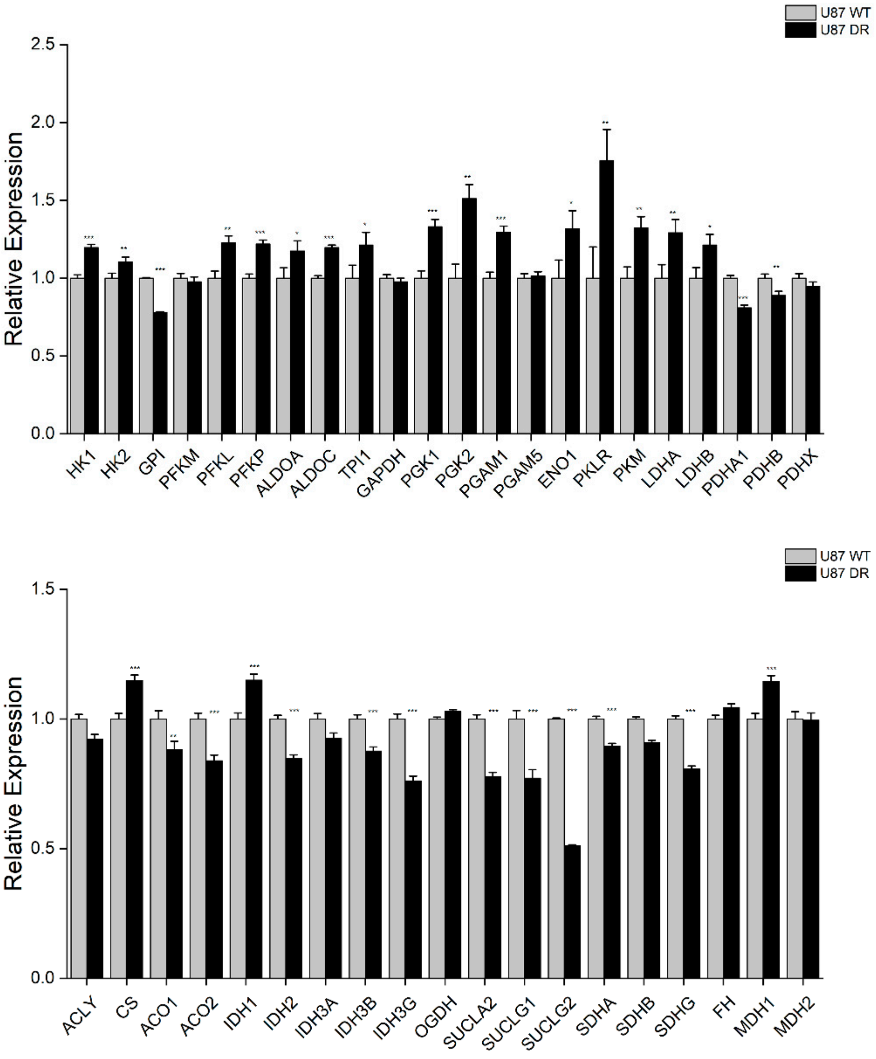

Figure S4. Relative expression of proteins involved in glycolysis and TCA cycle. (a) The expression of proteins involved in glycolysis were higher in U87 DR cells; (b) The expression of lots of proteins involved in TCA cycle were reduced in U87 DR cells. Significance was calculated using Student's t-test. ***: $p < 0.001$, **: $p < 0.01$, *: $p < 0.05$; n = 3, mean ± SD.

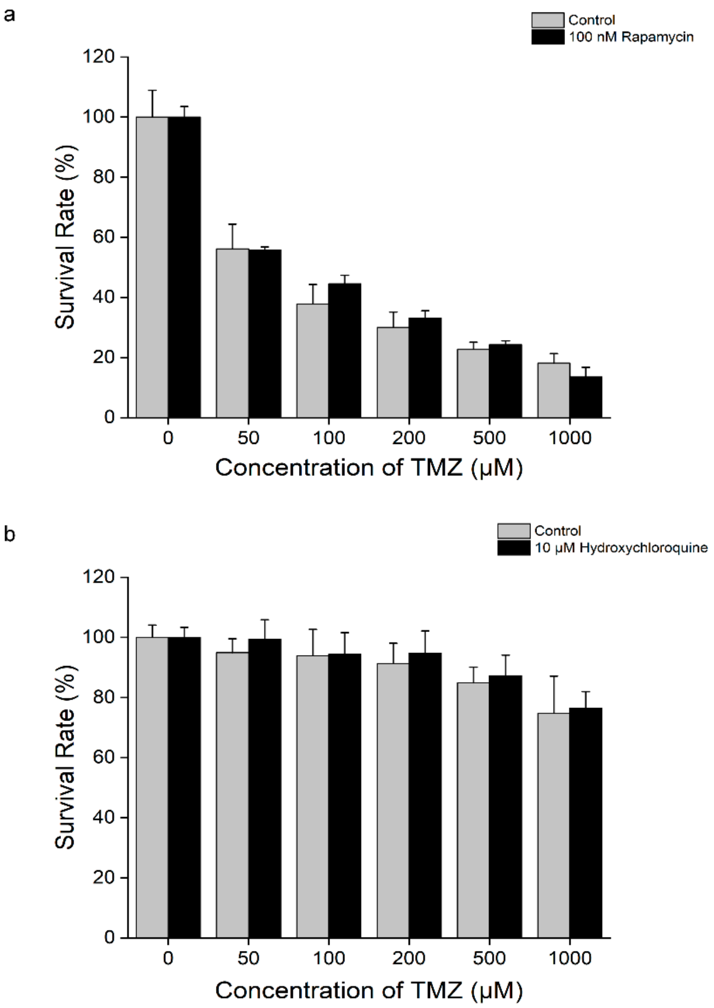

Figure S5. No evident correlation was observed between autophagy and TMZ-resistance in U87 DR cells. (a) Cell viability of U87 WT cells treated with 100 nM rapamycin and different dozes of TMZ was examined by CCK-8 assay; (b) Cell viability of U87 WT cells treated with 10 μM hydroxychloroquine and different dozes of TMZ was examined by CCK-8 assay.

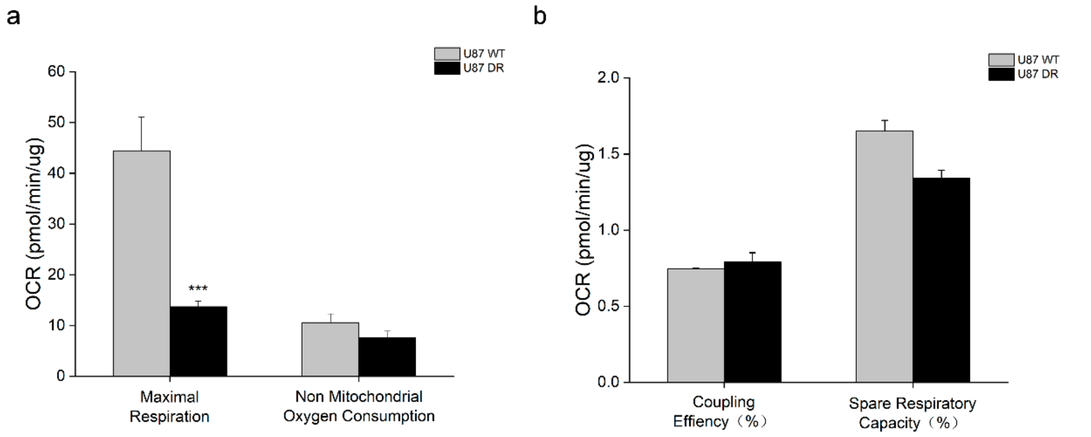

Figure S6. Mitochondrial respiration analysis in U87 DR cells. (a) Maximal respiration and Non mitochondrial oxygen consumption; (b) Coupling effiency(%) and Spare respiratory capacity(%). Significance was calculated using Student's t-test. ***: $p < 0.001$; n = 3, mean ± SD.

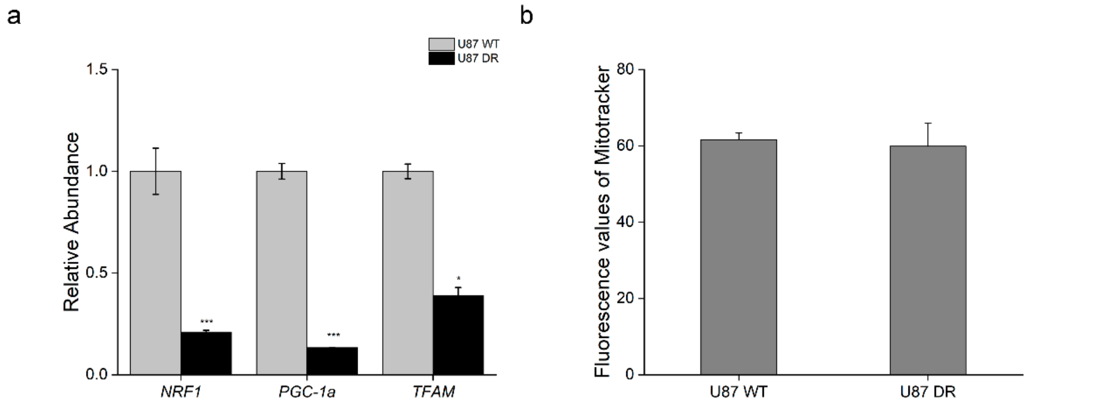

Figure S7. (a) Relative abundance of *NRF1*, *PGC-1α* and *TFAM* were reduced significantly in U87 DR cells; (b) Mitotracker staining was performed to detect the number of mitochondria. Significance was calculated using Student's t-test. ***: $p < 0.001$, *: $p < 0.05$; n = 3, mean ± SD.

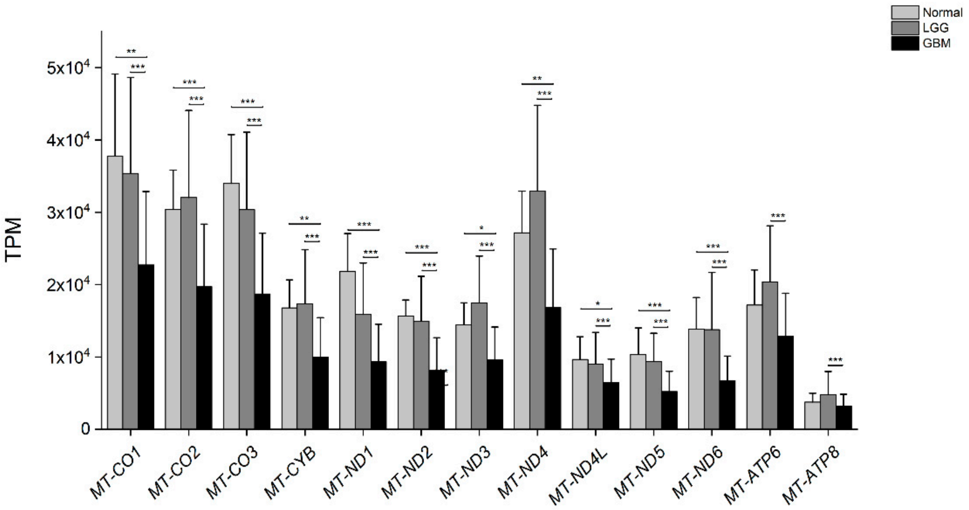

Figure S8. mRNA levels of protein-coding genes in mitochondrial genome in normal tissues (n=5), LGG tissues (n=534) and GBM tissues (n=170) were analyzed using TCGA transcriptome dataset. Significance was calculated using Student's t-test. ***: p < 0.001, **: p < 0. 01, *: p < 0.05; n = 3, mean ± SD.

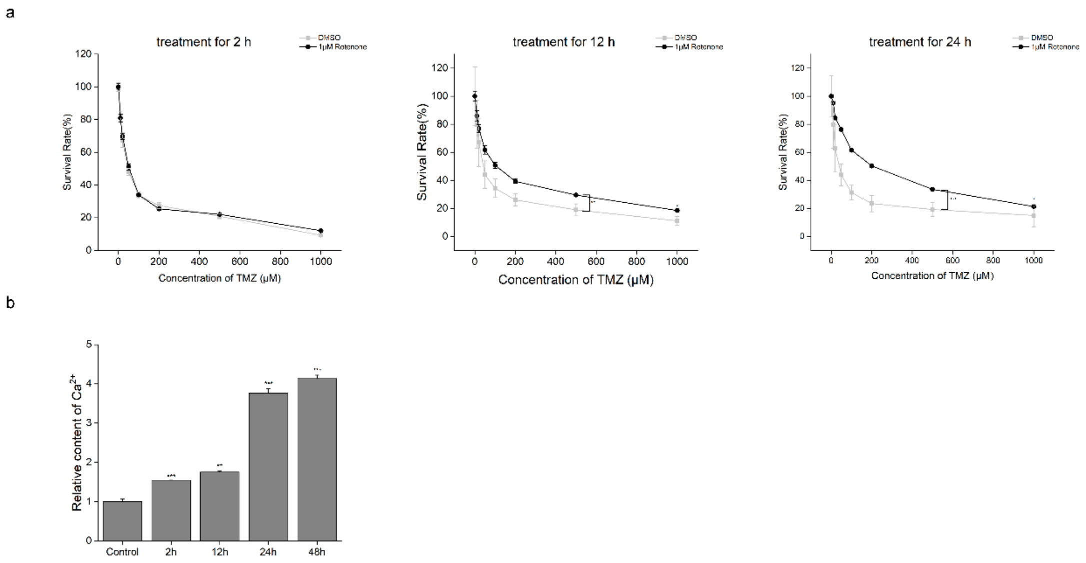

Figure S9. (a) Cell viability of U87 WT cells with rotenone treatment for different time (2 h, 12 h and 24 h), followed by different concentrations of TMZ treatment for additional 48 h was examined by CCK-8 assay.; (b) Relative content of free cytosolic Ca2+ was detected in U87 WT cells with rotenone treatment for different time. Significance was calculated using Student's t-test. ***: p < 0.001, **: p < 0.01, *: p < 0.05; n = 3, mean ± SD.
