# Supplementary material for: Multi-Omics Analyses Reveal Mitochondrial Dysfunction Contributing to Temozolomide Resistance in Glioblastoma Cells
Source: Biomolecules. 2023 Sep 19;13(9):1408. doi: 10.3390/biom13091408 (PMC10526285; doi:10.3390/biom13091408)
Supplement: Supplementary file 1 [file biomolecules-13-01408-s001.zip › Original Images for Blots.zip!/Original Images for Blots/Original Images for Blots.pdf]

Figure 2c

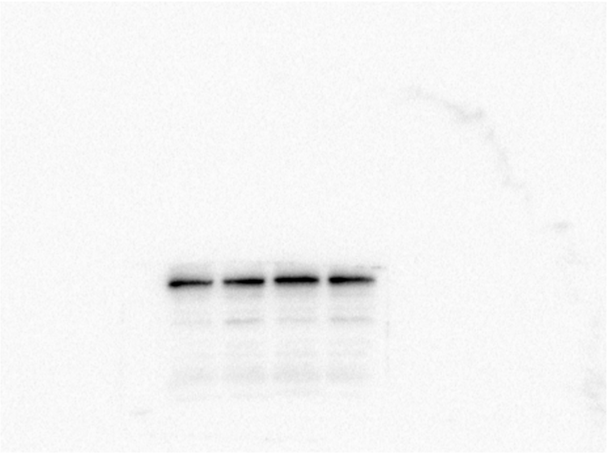

pro-caspase3

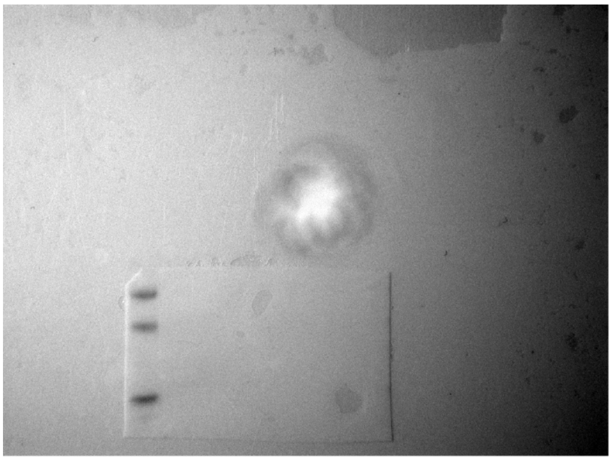

pro-caspase3 bright field

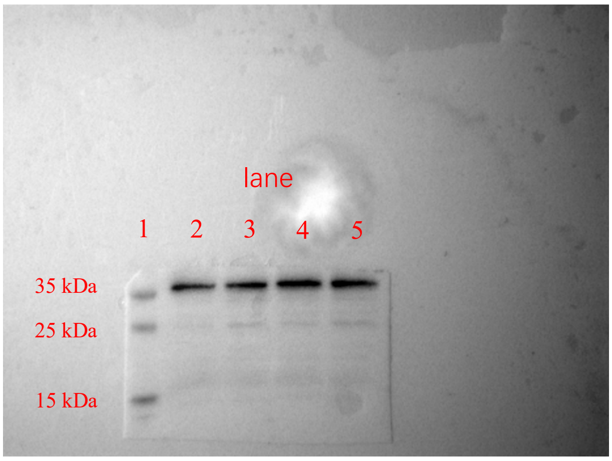

pro-caspase3 merge

lane1: protein marker (Thermo Fisher, Cat.No.: 26617)

lane2 and lane3: data for other project

lane4: U87 WT cells treated with 200 μM TMZ

lane5: U87 DR cells treated with 200 μM TMZ

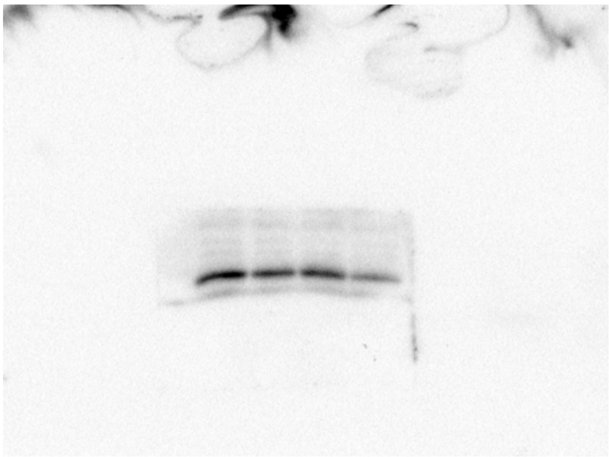

cleaved caspase 3

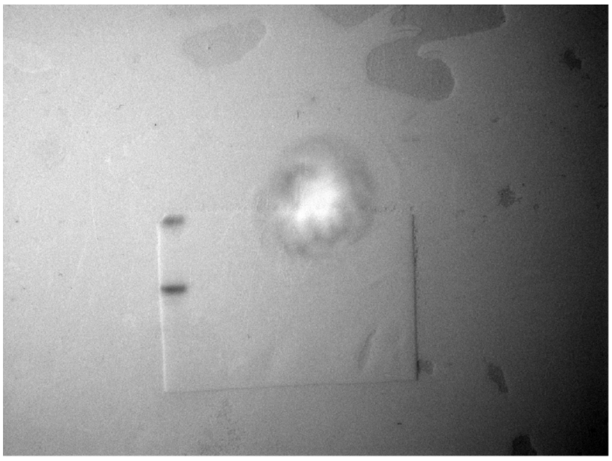

cleaved caspase 3 bright field

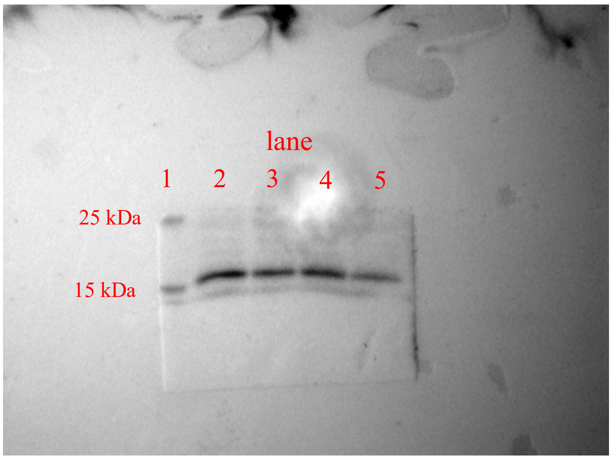

cleaved caspase3 merge

lane1: protein marker (Thermo Fisher, Cat.No.: 26617)

lane2 and lane3: data for other project

lane4: U87 WT cells treated by 200 µM TMZ

lane5: U87 DR cells treated by 200 µM TMZ

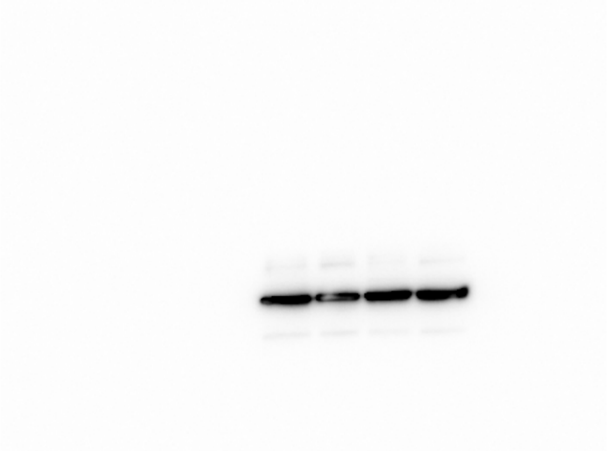

β-actin

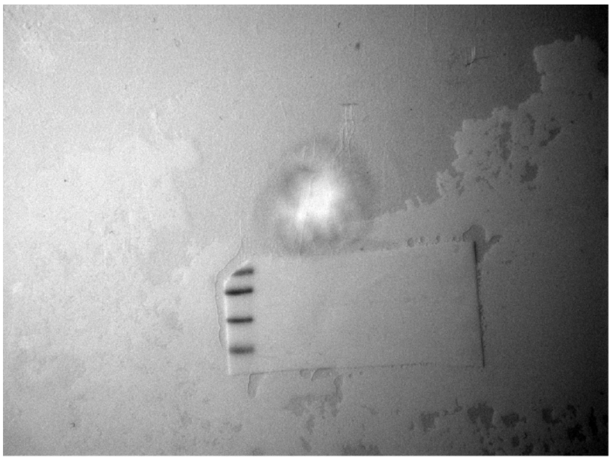

β-actin bright field

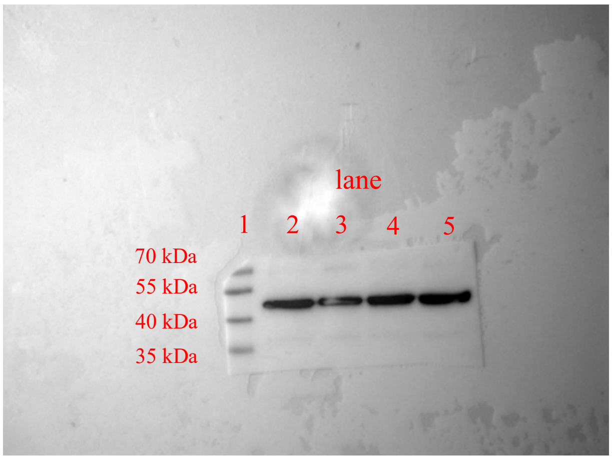

β-actin merge

lane1: protein marker (Thermo Fisher, Cat.No.: 26617)

lane2 and lane3: data for other project

lane4: U87 WT cells treated by 200 μM TMZ

lane5: U87 DR cells treated by 200 μM TMZ

Figure 6b

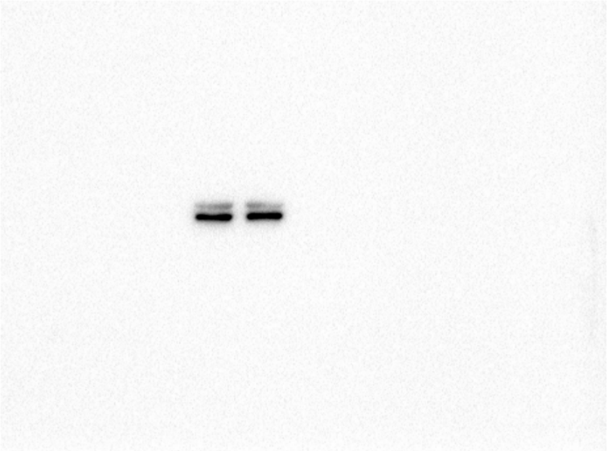

p-MAPK

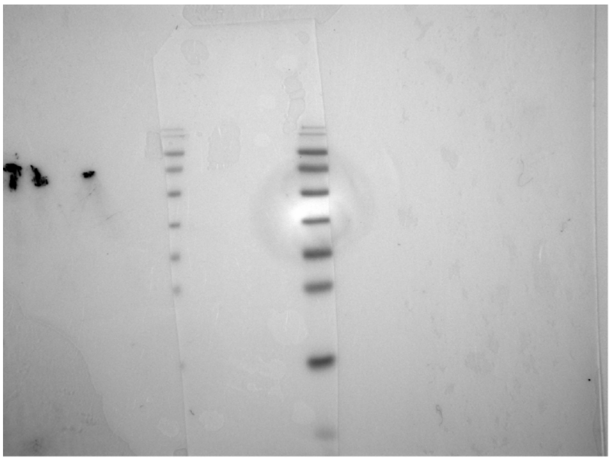

p-MAPK bright field

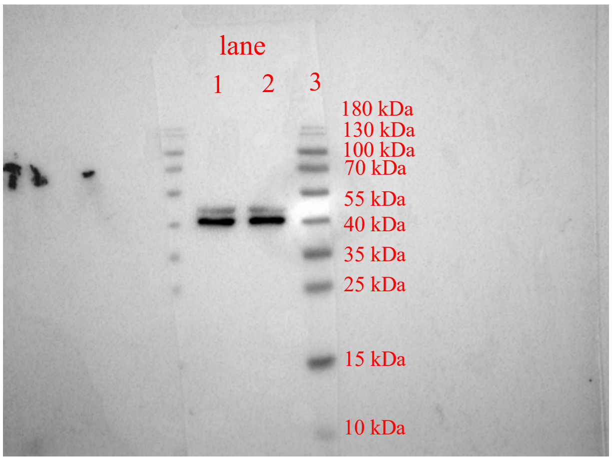

p-MAPK merge

lane1: U87 WT cells

lane2: U87 DR cells

lane3: protein marker (Thermo Fisher, Cat.No.: 26617)

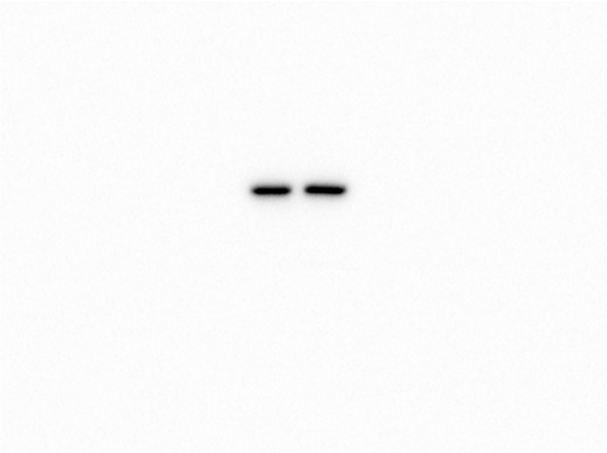

MAPK

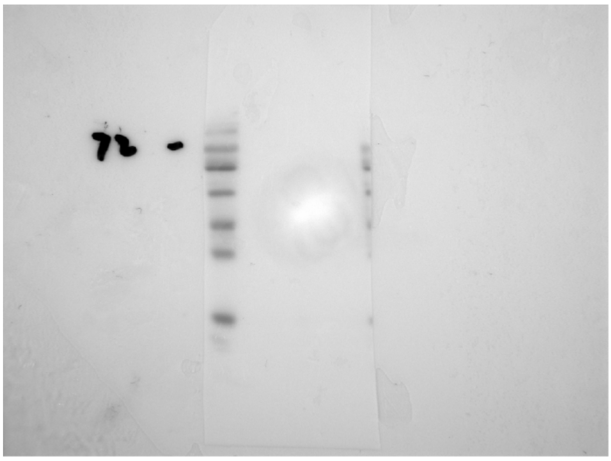

MAPK bright field

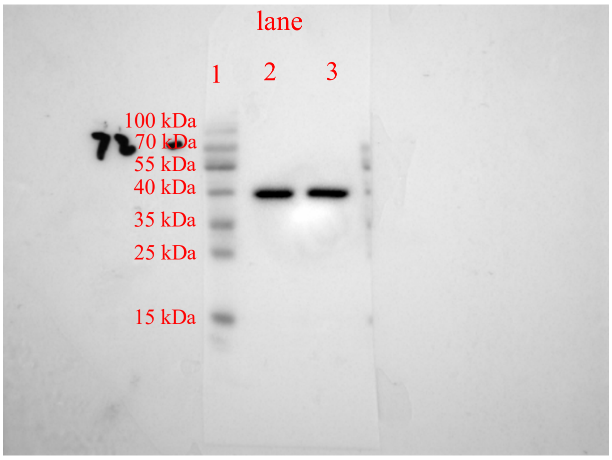

p-MAPK merge

lane1: protein marker (Thermo Fisher, Cat.No.: 26617)

lane2: U87 WT cells

lane3: U87 DR cells

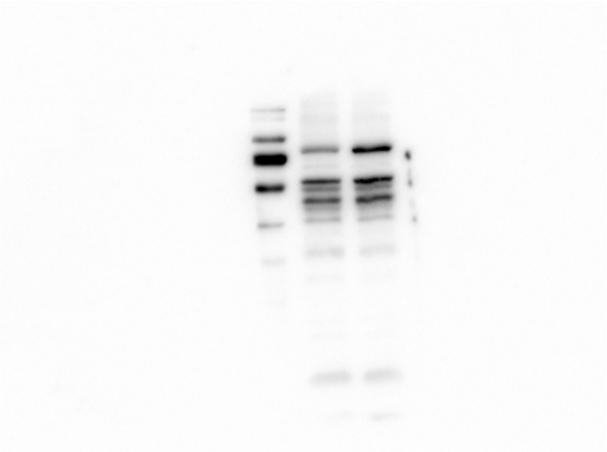

p-JNK (Thr183/Tyr185)

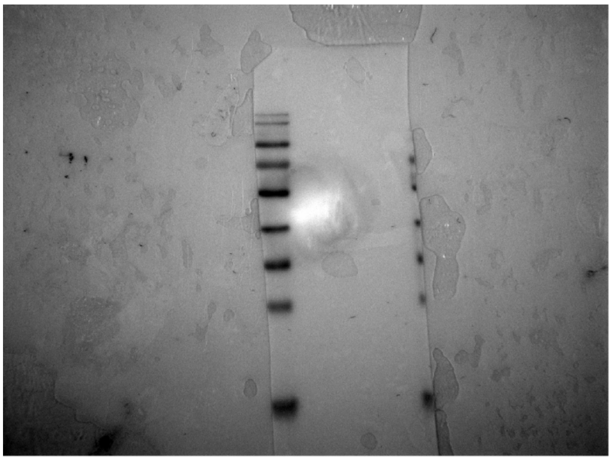

p-JNK (Thr183/Tyr185) bright field

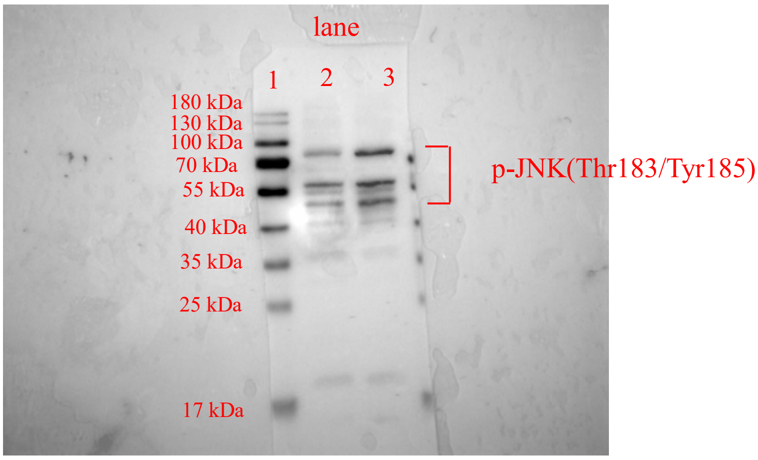

p-JNK (Thr183/Tyr185) merge

lane1: protein marker (Thermo Fisher, Cat.No.: 26617)

lane2: U87 WT cells

lane3: U87 DR cells

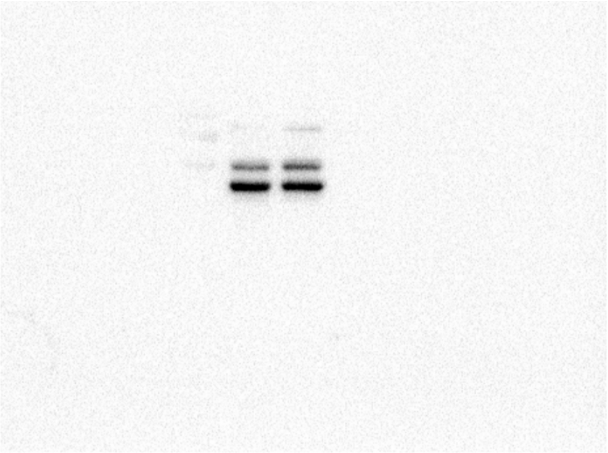

JNK

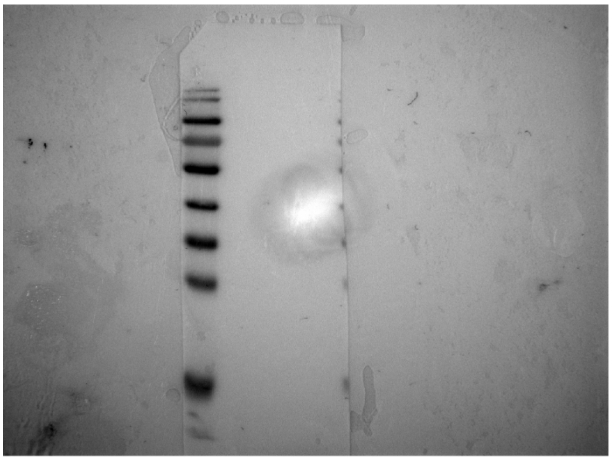

JNK bright field

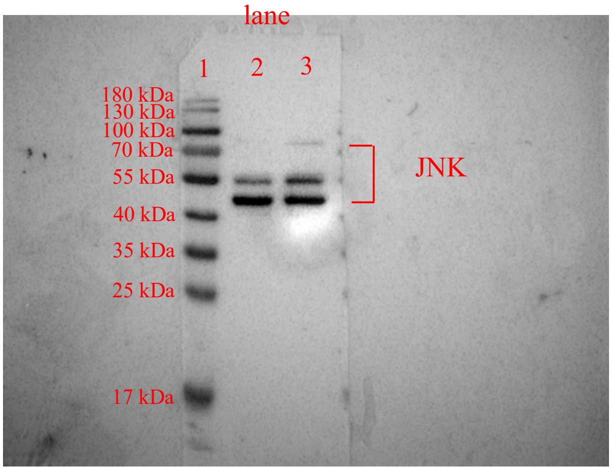

JNK merge

lane1: protein marker (Thermo Fisher, Cat.No.: 26617)

lane2: U87 WT cells

lane3: U87 DR cells

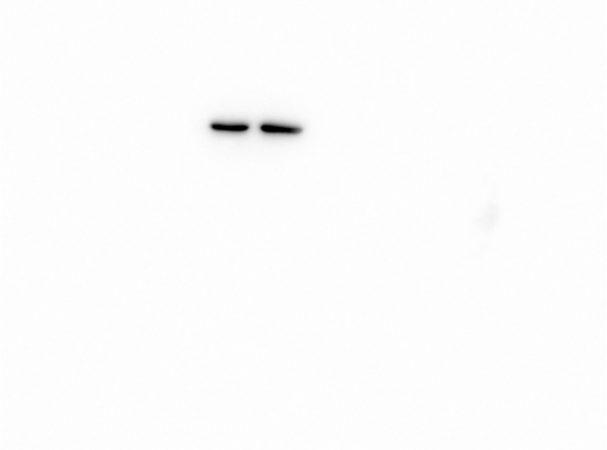

β-actin

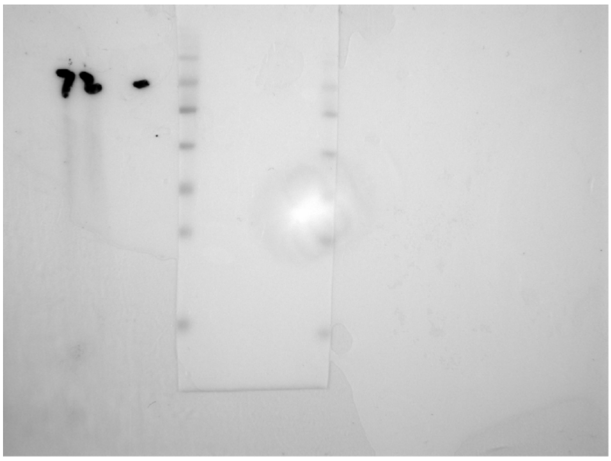

β-actin bright field

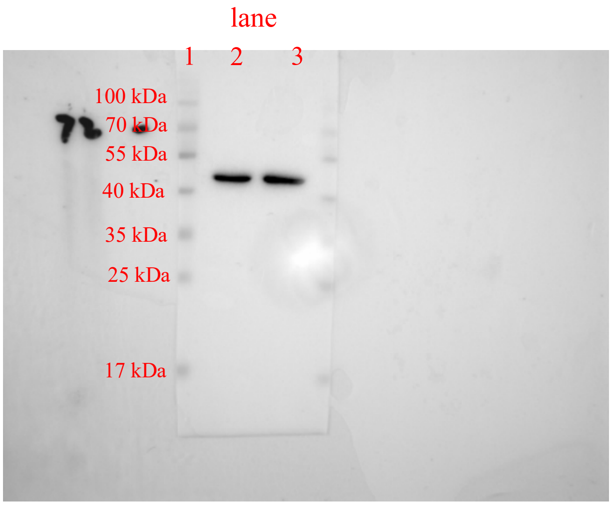

β-actin merge

lane1: protein marker (Thermo Fisher, Cat.No.: 26617)

lane2: U87 WT cells

lane3: U87 DR cells

Figure 6d

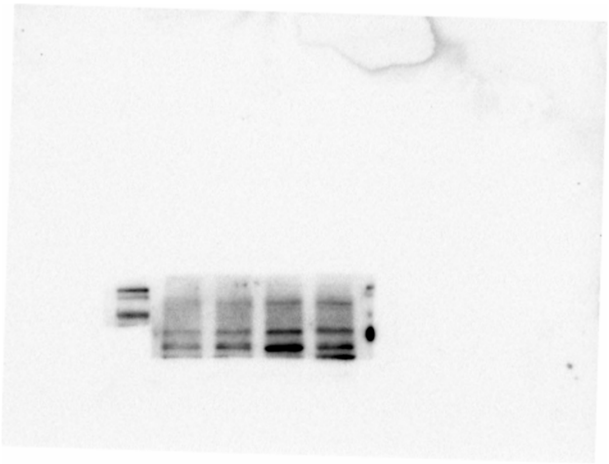

p-STAT3 (S727)

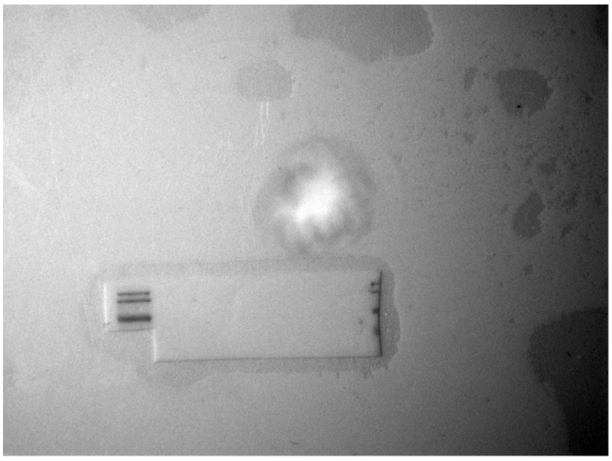

p-STAT3 (S727) bright field

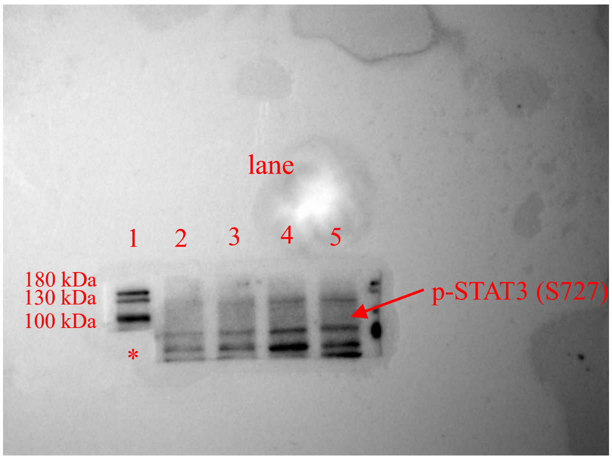

p-STAT3 (S727) merge

lane1: protein marker (Thermo Fisher, Cat.No.: 26617)

lane2: U87 WT cells

lane3: U87 DR cells

lane4 and lane5: data for other project

*: Our ECL kit could react with protein marker at 70 kDa occasionally. It formed a bright black dot at this PVDF membrane and interfered the results. Therefore, we cut the PVDF membrane at 70 kDa in lane 1.

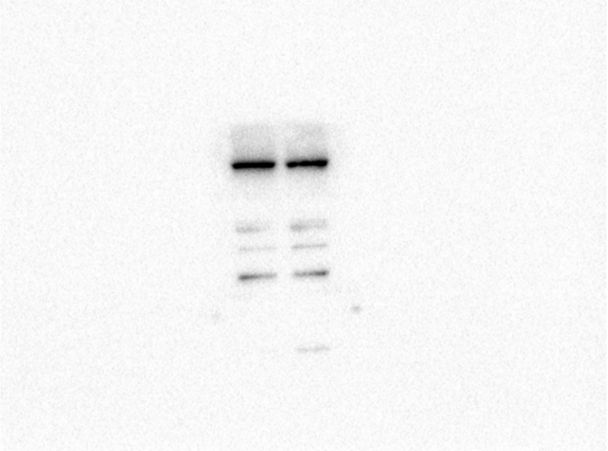

STAT3

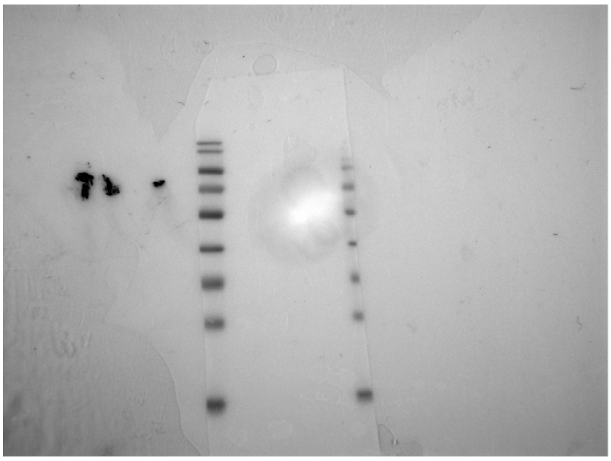

STAT3 bright field

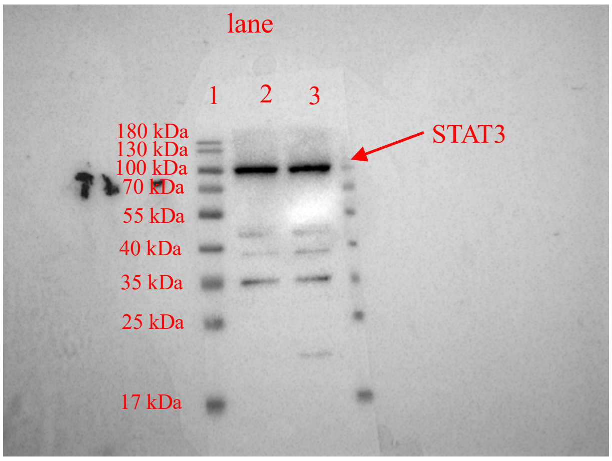

STAT3 merge

lane1: protein marker (Thermo Fisher, Cat.No.: 26617)

lane2: U87 WT cells

lane3: U87 DR cells

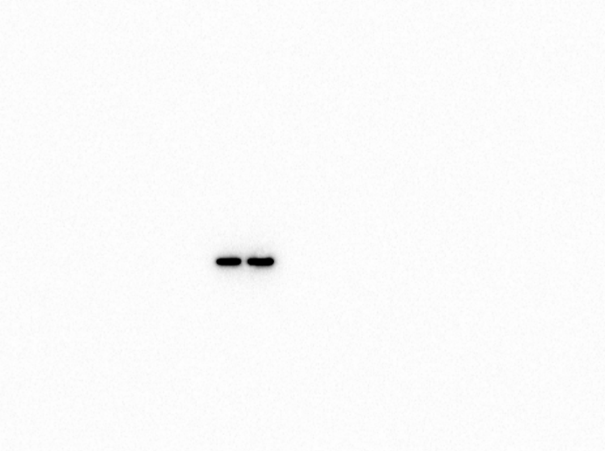

β-actin

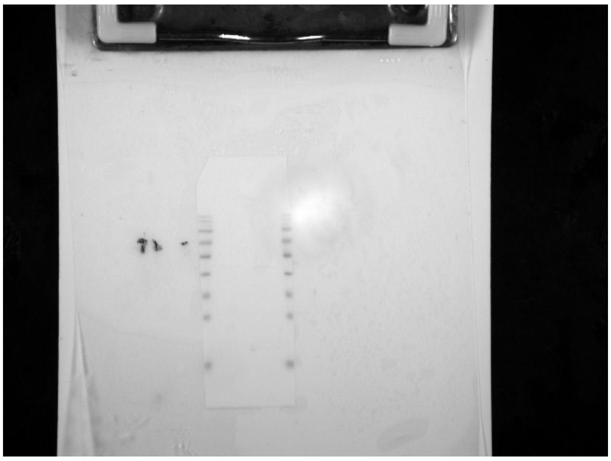

β-actin bright field

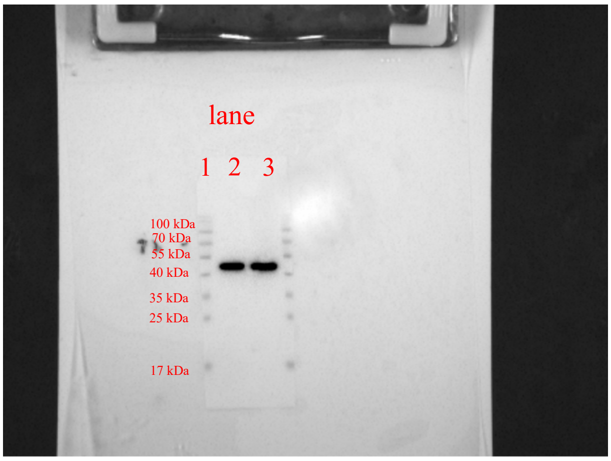

β-actin merge
lane1: protein marker (Thermo Fisher, Cat.No.: 26617)
lane2: U87 WT cells
lane3: U87 DR cells

Figure 6e

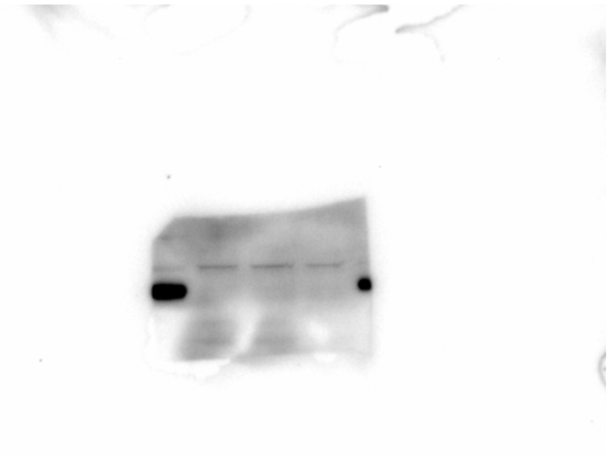

p-STAT3 (S727)

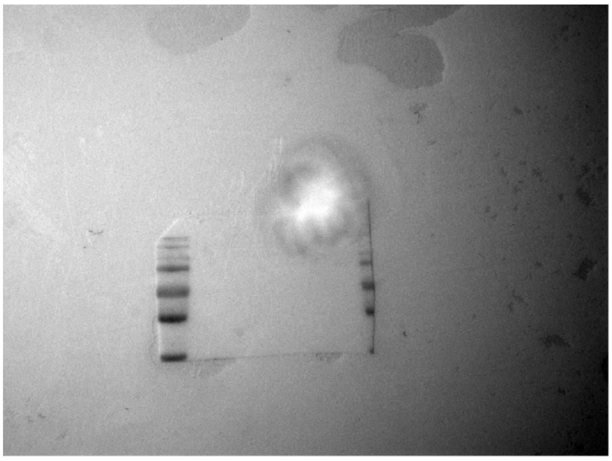

p-STAT3 (S727) bright field

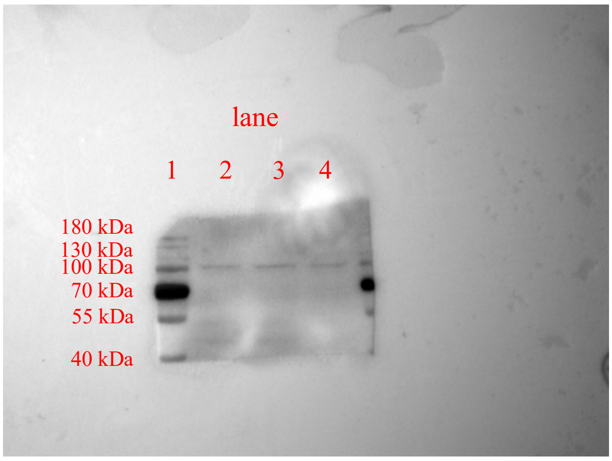

p-STAT3 (S727) merge

lane1: protein marker (Thermo Fisher, Cat.No.: 26617)

lane2: U87 DR cells treated with DMSO

lane3: U87 DR cells treated with 0.1 µM 600125

lane4: U87 DR cells treated with 1 µM 600125

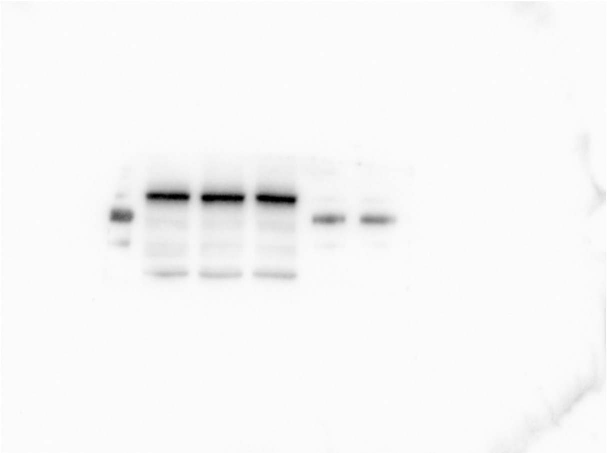
STAT3

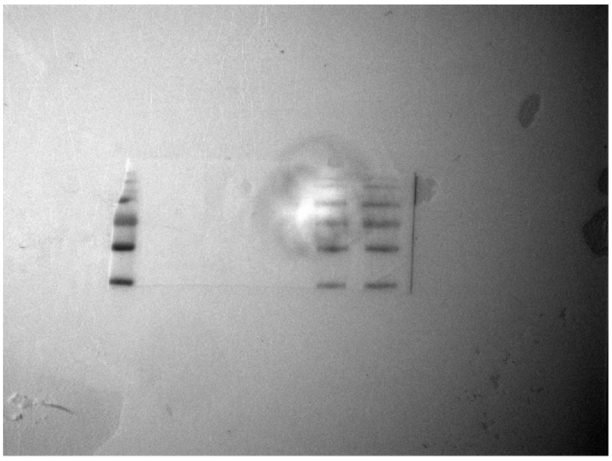
STAT3 bright field

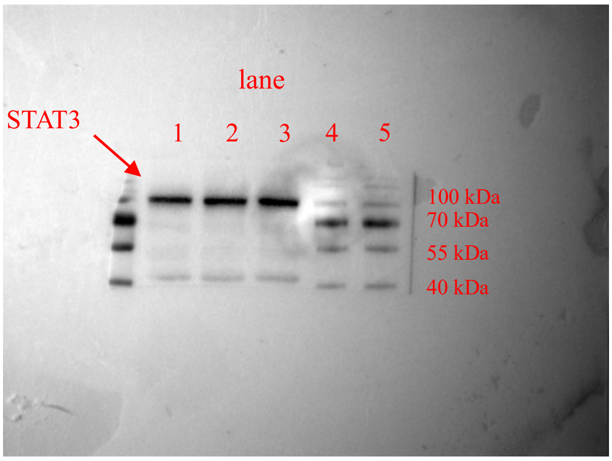

STAT3 merge
lane1: U87 DR cells treated with DMSO
lane2: U87 DR cells treated with 0.1 μM 600125
lane3: U87 DR cells treated with 1 μM 600125
lane4: protein marker (Thermo Fisher, Cat.No.: 26617)
lane5: protein marker (Thermo Fisher, Cat.No.: 26617)

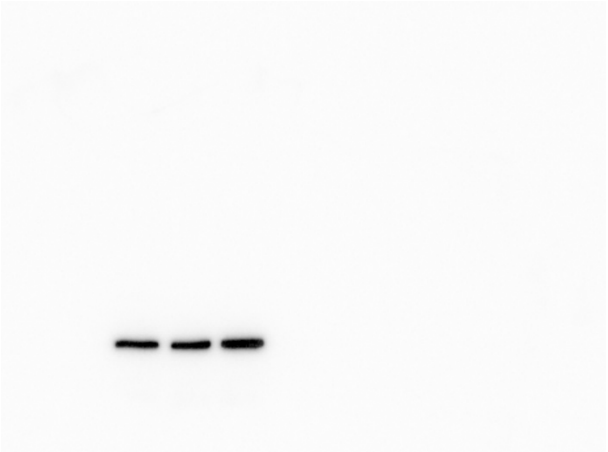
β-actin

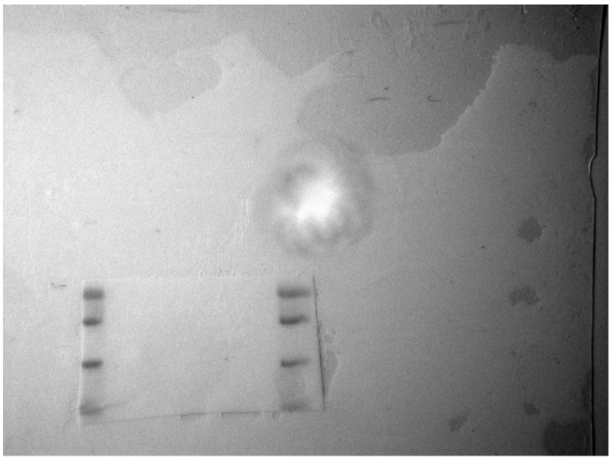
β-actin bright field

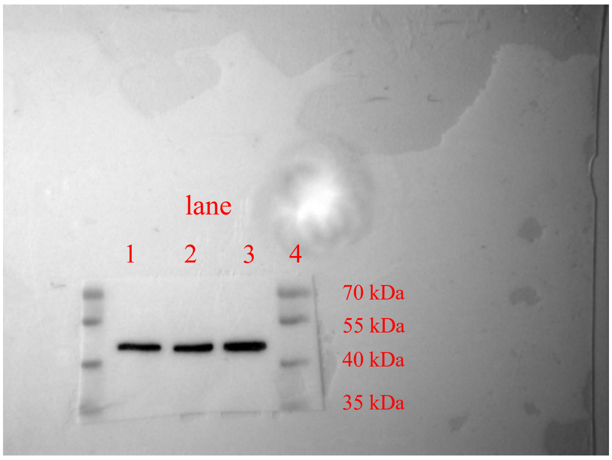

β-actin merge

lane1: U87 DR cells treated with DMSO

lane2: U87 DR cells treated with 0.1 μM 600125

lane3: U87 DR cells treated with 1 μM 600125

lane4: protein marker (Thermo Fisher, Cat.No.: 26617)
